# Genome Editing Technology and Its Application Potentials in the Industrial Filamentous Fungus *Aspergillus oryzae*

**DOI:** 10.3390/jof7080638

**Published:** 2021-08-05

**Authors:** Jun-ichi Maruyama

**Affiliations:** 1 Department of Biotechnology, The University of Tokyo, Bunkyo-ku, Tokyo 113-8657, Japan; amarujun@g.ecc.u-tokyo.ac.jp; Tel.: +81-3-5841-5164; 2 Collaborative Research Institute for Innovative Microbiology, The University of Tokyo, Bunkyo-ku, Tokyo 113-8657, Japan

**Keywords:** *Aspergillus oryzae*, genome editing, TALENs, CRISPR/Cas9, brewing, heterologous production

## Abstract

*Aspergillus oryzae* is a filamentous fungus that has been used in traditional Japanese brewing industries, such as the sake, soy sauce, and *miso* production. In addition, *A. oryzae* has been used in heterologous protein production, and the fungus has been recently used in biosynthetic research due to its ability to produce a large amount of heterologous natural products by introducing foreign biosynthetic genes. Genetic manipulation, which is important in the functional development of *A. oryzae*, has mostly been limited to the wild strain RIB40, a genome reference suitable for laboratory analysis. However, there are numerous industrial brewing strains of *A. oryzae* with various specialized characteristics, and they are used selectively according to the properties required for various purposes such as sake, soy sauce, and *miso* production. Since the early 2000s, genome editing technologies have been developed; among these technologies, transcription activator-like effector nucleases (TALENs) and clustered regularly interspaced short palindromic repeats/CRISPR-associated protein 9 (CRISPR/Cas9) have been applied to gene modification in *A. oryzae*. Notably, the CRISPR/Cas9 system has dramatically improved the efficiency of gene modification in industrial strains of *A. oryzae*. In this review, the development of genome editing technology and its application potentials in *A. oryzae* are summarized.

## 1. Introduction

*Aspergillus oryzae* is a filamentous fungus that has been used in traditional Japanese brewing industries, such as the sake, soy sauce, and *miso* production [1]. *A. oryzae* has also been used in heterologous protein production due to its ability to secrete a large amount of proteins into medium and its guaranteed safety from its long-term use in food production [2]. Furthermore, although *A. oryzae* is a clean host that produces almost no secondary metabolites, it has recently been used in biosynthetic research since the fungus can produce a large amount of heterologous natural products by introducing foreign biosynthetic enzyme genes [3].

Whole genome sequencing of *A. oryzae* has revealed that it has a genome size of 37.9 Mb with eight chromosomes and more than 12,000 genes [4]. In the genetic manipulation of *A. oryzae*, host vector systems based on various auxotrophic markers have been prepared [5], but the efficiency of gene deletion was initially low. Gene deletion efficiency was dramatically improved by the deficiency of Ku70/Ku80 and LigD, which are involved in non-homologous end joining (NHEJ) [6,7], and the functional development in *A. oryzae* by genetic engineering has progressed. In heterologous protein production using *A. oryzae*, for example, useful proteins derived from animals and plants were produced [8,9,10,11,12], and several strains that produce high amounts of heterologous proteins have been developed by manipulating genes related to protein secretion transport [13,14,15] and by multiple deletions of 10 protease genes [16,17] based on transformation marker recycling [18,19].

Such genetic manipulation of *A. oryzae* was initially limited to the wild strain RIB40, a genome reference which is suitable for laboratory analysis [4]. However, there are numerous industrial brewing strains of *A. oryzae* with various characteristics, which are used selectively according to the properties required for various purposes such as sake, soy sauce, and *miso* production. Furthermore, in enzyme production, high-producing strains obtained by repeating mutation treatments have been used in industry. Genetic manipulation techniques are required for genetic analysis of the characteristics in these industrial strains and for further breeding. However, a lot of labor and time are required to prepare the host vector system for each industrial strain and to delete the factors involved in NHEJ. In addition, as the gene deletion efficiency of the *A. oryzae* industrial strains used to be extremely low, it was difficult to apply gene manipulation to industrial purposes.

**Figure 1:**
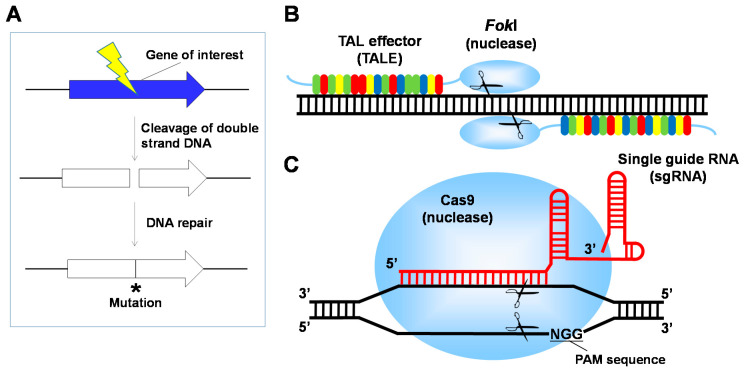
Genome editing technologies applied to *A. oryzae*. (**A**) Basic principles of genome editing. The engineered nuclease is targeted to efficiently generate DNA double-strand breaks, which activates the DNA repair pathways and then introduces a site-specific mutation. (**B**) Transcription activator-like effector nucleases (TALENs). TALENs recognize two target sites separated with a 12–20 bp spacer sequence cleaved by *Fok*I nuclease domain. (**C**) CRISPR/Cas9 system. Cas9 nuclease is recruited by single guide RNA (sgRNA) to the target DNA sequence located immediately upstream of the protospacer-associated motif (PAM) sequence. * site-specific mutation.

Since the early 2000s, genome editing technologies have been developed in which engineered nucleases induce DNA double-strand breaks at the target sites on genomes, and mutations are introduced during the DNA repair process (Figure 1A) [20]. Since it is possible to efficiently modify genes in a wide range of organisms such as animals and plants, the technologies are expected to be widely applied for medical and agricultural purposes [21,22]. Additionally, this genome editing technology has dramatically improved gene modification efficiency in industrial strains of *A. oryzae*, and the methodology for industrial breeding has been changing substantially. In this review, the development of genome editing technology and its applications to functional development in *A. oryzae* are summarized.

## 2. Genome Editing Using TALENs in *A. oryzae*

Zinc-finger nuclease (ZFN), which was first developed as a genome editing technology, is a fusion protein consisting of a DNA-binding domain and a nuclease domain [23]. For the DNA-binding domain, a zinc finger domain that binds to a specific 3-base sequence is used. The double-stranded DNA is cleaved by the sequence-independent nuclease domain derived from the restriction enzyme *Fok*I, and then a mutation is introduced [23]. Furthermore, to improve the specificity for the base sequence, transcription activator-like effector nucleases (TALENs) were developed as the second generation of genome editing. TALENs are fusion proteins consisting of the *Fok*I nuclease domain and multiple arrays of DNA-binding domains from transcription factor-like effector (TAL effector; TALE) proteins derived from bacteria of the genus *Xanthomonas* (Figure 1B) [24]. TALE, a DNA-binding unit with 34 amino acids, recognizes a single base pair, which is determined by the 12th and 13th variable amino acids. In *A. oryzae*, highly active platinum-fungal TALENs successfully introduced mutations into target genes with an efficiency of >95% in *A. oryzae* wild strain RIB40 [25]. When targeting the *sC* gene encoding ATP sulfurylase conferring sulfate assimilation, various mutation patterns were obtained, revealing deletions from several bp to over 1.0 kb and insertions around the target sequence. The ratio of the large deletions was decreased by the deficiency of LigD involved in NHEJ repair [25]. Thus, genome editing using TALENs mainly caused large deletions at the target region with high efficiency in *A. oryzae*.

## 3. Genome Editing Using the CRISPR/Cas9 System in *A. oryzae*

Among the genome editing technologies, the clustered regularly interspaced short palindromic repeats/CRISPR-associated protein 9 (CRISPR/Cas9) system is an efficient cost-effective method employed in a wide variety of organisms such as animals and plants; it is expected to be widely applied to therapeutic treatment and agriculture [26]. The CRISPR/Cas9 system mainly uses two components, Cas9 nuclease derived from the bacteria *Streptococcus pyogenes* and single guide RNA (sgRNA). Firstly, Cas9 is recruited to the target (protospacer) sequence by sgRNA that complementarily binds to the target DNA sequence on the genome; it recognizes the protospacer-associated motif (PAM) sequence (NGG) that is placed immediately after the target sequence (Figure 1C). Subsequently, Cas9 causes DNA double-strand breaks in the target region, and then a mutation is introduced as error during the process of DNA repair.

**Figure 2:**
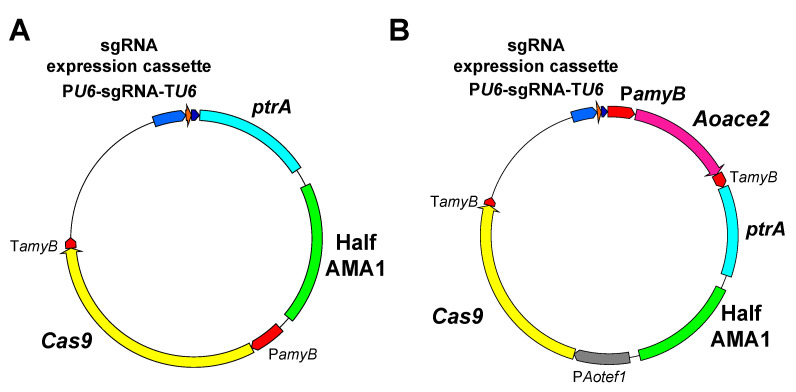
Genome-editing plasmids of the CRISPR/Cas9 system in *A. oryzae*. (**A**) Autonomous replication plasmid for genome editing. (**B**) Recyclable autonomous replication plasmid for genome editing. Expression of the *Aoace2* gene is regulated by the conditional promoter P*amyB*. *ptrA*: Pyrithiamine resistance gene, P*amyB* and T*amyB*: Promoter and terminator of α-amylase gene, respectively. P*tef1*: Constitutive promoter of the gene for translation elongation factor 1α.

In 2016, the CRISPR/Cas9 system was first applied to targeted mutagenesis in *A. oryzae* [27]. To express the two CRISPR/Cas9 components, Cas9 and sgRNA, genome-editing plasmids are often constructed in filamentous fungi [28]. To express Cas9 in *A. oryzae*, its encoding DNA was codon-optimized and the nuclear localization signals SV40 NLSs were fused to both the N- and C-terminal ends for nuclear localization. For sgRNA expression, the promoter and terminator, of which transcription is dependent on RNA polymerase III, are used to avoid the attachment of a cap structure and poly-A tail to the sgRNAs [29]. In *A. oryzae*, sgRNA containing the target sequence of 20 bases is expressed under the control of the promoter and terminator of the *U6* gene encoding the small nuclear RNA (Figure 2A,B) [27]. The *U6* promoter from *Aspergillus niger* was compatibly applied to the functional expression of sgRNA in *A. oryzae* [30], and the use of tRNA promoters was also reported for sgRNA expression in other filamentous fungal species [31]. Additionally, two ribozyme sequences consisting of 3′-end hepatitis delta virus and 5′-end hammerhead enable functional sgRNA expression in filamentous fungi [28].

**Figure 3:**
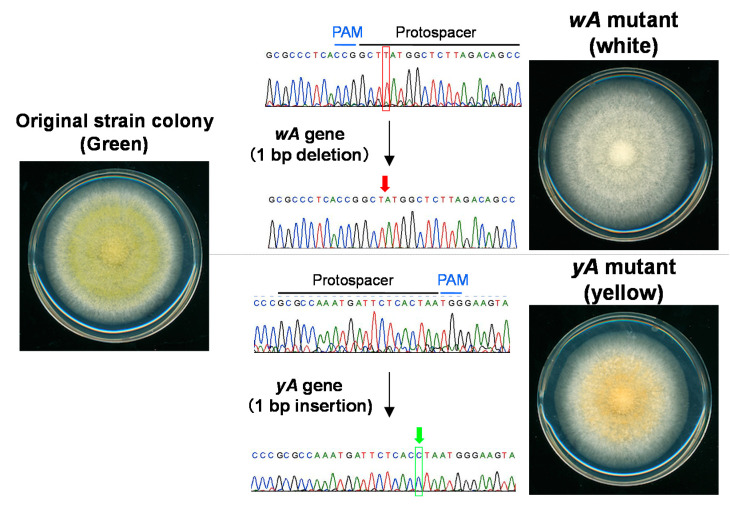
Example of mutation introduction by the CRISPR/Cas9 system in *A. oryzae*. *A. oryzae* colonies are typically green due to pigment synthesis in conidia. Cas9 nuclease was targeted to protospacer regions flanking the protospacer-associated motif (PAM) sequence (NGG) within the *wA* and *yA* genes involved in pigment synthesis, resulting in a 1-bp deletion and insertion, respectively. Gene inactivation changed the colony color to white or yellow.

When using the CRISPR/Cas9 system in filamentous fungi, AMA1 derived from *Aspergillus nidulans* for autonomous plasmid replication is generally contained in the genome-editing plasmid [28]. In *A. oryzae*, a half region of the DNA fragment AMA1 was included in the genome-editing plasmid (Figure 2A,B), and mutation efficiency using the autonomous replicating plasmid was significantly improved up to 50–100% [32] compared with the mutation ratios (10–20%) when integrating the genome-editing plasmid into the genome [27]. For example, by targeting the genes *wA* and *yA* involved in the conidial pigment synthesis of *A. oryzae*, of which the colony color is normally green, it is possible to obtain mutants that form white or yellow spores (Figure 3). Notably in *A. oryzae*, mutations were successfully introduced not only in the wild strain RIB40 but also in industrial strains [27,32]. Therefore, the CRISPR/Cas9 system has succeeded in efficient gene modification in industrial strains of *A. oryzae*, which had been quite difficult to genetically manipulate in the past, without the need to construct any host/vector systems.

Besides the plasmid-mediated transformation for expressing Cas9 and sgRNA, a method employing the direct introduction of the Cas9/sgRNA ribonucleoprotein complex has also been performed for genome editing in filamentous fungi [33]. In *A. oryzae*, a novel pyrithiamine resistance marker, the putative thiamine transporter gene *thiI*, was found by whole genome sequencing of a newly obtained pyrithiamine-resistant mutant [34]. In the CRISPR/Cas9 system, by introducing the Cas9/sgRNA ribonucleoprotein complex targeting the *thiI* gene, pyrithiamine-resistant mutants were successfully obtained, which did not confer any auxotrophies. Moreover, simultaneous introduction of ribonucleoprotein complexes targeting *thiI* and another gene generated double mutants under the selection for pyrithiamine resistance, and the mutation frequency was 5.5–8.2% [34]. Collectively, loss-of-function mutation in the *thiI* gene can be a marker gene for generating mutants of targeted genes in *A. oryzae*.

## 4. Efficient Multiple-Gene Modification by Recycling the Genome-Editing Plasmid in *A. oryzae*

Industrial microorganisms are bred to acquire excellent properties, typically through multiple-step mutagenesis processes. One advantage of genome editing using the CRISPR/Cas9 system is that mutations can be introduced into multiple genes at once by simultaneously expressing sgRNAs that target different genes [29]. However, the mutation efficiency decreases as the number of genes to be modified at the same time increases. In addition, for modifying many genes in multiple steps, it is necessary to remove the introduced genome-editing plasmid each time.

**Figure 4:**
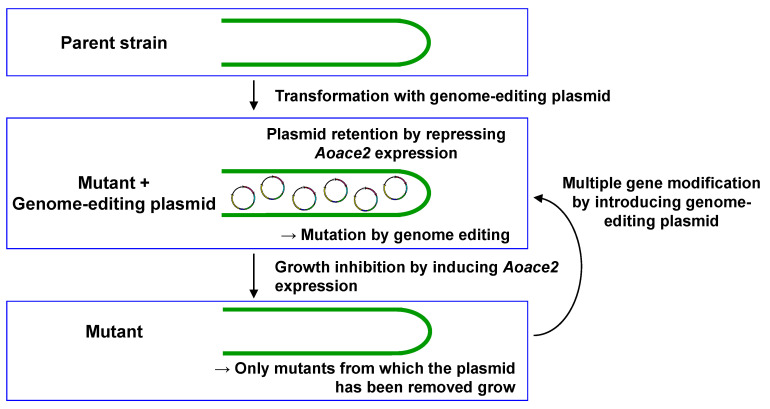
Multiple-gene modification by recycling the autonomous replication plasmid in *A. oryzae*. The parent strain is transformed with the genome-editing plasmid targeting a gene, and mutation is introduced while the plasmid retains under the *Aoace2*-repressed condition. Then, the plasmid is removed from the mutant growing against the growth inhibition by inducing *Aoace2* expression. Finally, further transformation with the genome-editing plasmid for next gene mutagenesis results in the multiple gene modification.

Therefore, a plasmid recycling technique for genome editing was established in *A. oryzae* with the aim of introducing multiple mutations in the same strain [32]. Since there are numerous industrial *A. oryzae* strains, a large amount of labor is needed to obtain an auxotrophic host from each strain. To avoid this, the drug resistance gene *ptrA* against pyrithiamine was used as a transformation marker (Figure 2A,B) [32]. Before this, there were no reports of forced recycling of autonomous replication plasmids in filamentous fungi, and no methods had been developed for reusing a drug resistance marker. For recycling an autonomously replicating genome-editing plasmid, conditional expression of *Aoace2*, of which overexpression was found to significantly inhibit growth, was employed [32]. The *Aoace2* gene under the control of the conditionally expressing promoter of the α-amylase gene *amyB* was inserted into the genome-editing plasmid (Figure 2B). The technique for modifying multiple genes by recycling the genome-editing plasmid is described as follows (Figure 4) [32]. First, the parent strain of *A. oryzae* is transformed to obtain a transformant that becomes resistant to pyrithiamine by the *ptrA* marker gene. The plasmid is retained when the expression of *Aoace2* is repressed, and the mutation is introduced by genome editing. Once the mutant strain is obtained, the genome-editing plasmid used to introduce the mutation is no longer needed. Then, *Aoace2* expression under the control of the conditional promoter *amyB* is induced, causing a growth inhibition. Only the cell, from which the plasmid has been removed, grows selectively, but at the same time, it becomes sensitive to pyrithiamine. The growing mutant can be transformed with another genome-editing plasmid that introduces a mutation into the next target gene. By repeating these procedures, multiple gene mutants can be obtained. Multiple gene modification by recycling the genome-editing plasmid can be applied not only to the wild strain RIB40 but also to industrial strains [32]. It should be noted that since the mutant in which the genome-editing plasmid has been removed has no foreign DNA remaining, it is possible to efficiently breed the strain indistinguishable from the naturally occurring mutant.

**Figure 5:**
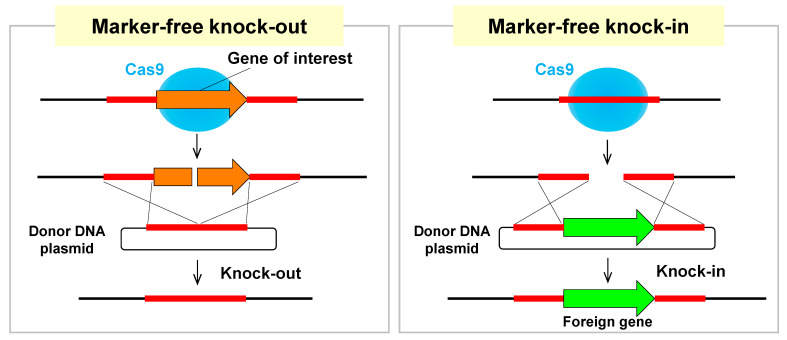
Marker-free knock-out/knock-in by the CRISPR/Cas9 system. Co-transformation with circular donor DNA and genome-editing plasmids enables efficient marker-free knock-out/knock-in via homologous recombination. When the donor DNA only contains flanking regions, the target gene can be deleted. When a foreign gene is contained between the flanking regions, the gene can be integrated at the target locus. Note that, in combination with recycling the genome-editing plasmid as shown in Figure 4, the marker-free knock-out/knock-in techniques enable to unlimitedly repeat multiple gene modifications.

Furthermore, by simultaneously introducing the donor DNA together with the genome-editing plasmid, homologous recombination at the target site can be performed efficiently [32]. In other words, the use of donor DNA enables a knock-out that deletes the target gene and a knock-in that integrates a foreign gene into the target site (Figure 5). Surprisingly, marker-free genetic engineering can be efficiently performed without inactivating the NHEJ pathway, such as deficiencies of Ku70/80 and LigD. Here, a transformation marker is used for the genome-editing plasmid, but highly efficient knock-in/knock-out is possible without integrating the transformation marker at the target site. Generally, it has been common to integrate the transformation marker at the target site, but the high efficiency of genome editing technology facilitates marker-free knock-out/knock-in, which completely changes the conventional concept of gene modification.

Genome-editing plasmid recycling and marker-free knock-out/knock-in technologies have allowed gene modifications to be repeated indefinitely without a limitation for the number of transformation markers.

## 5. Efficient Gene Modification Using Genome Editing for Heterologous Production by *A. oryzae*

As previously mentioned, genome editing with the CRISPR/Cas9 system enables the efficient modification of genes in *A. oryzae*, and heterologous production is an effective application. In particular, the use of genome editing in the production of heterologous natural products using *A. oryzae* has recently been progressing. The quadruple auxotrophic strain NSAR1 has mainly been used as a host in the heterologous production of natural products for the purpose of biosynthetic research using *A. oryzae* [3,5]. Secondary metabolic gene clusters are composed of many genes to synthesize natural products with complex chemical structures, but all these genes must be introduced into one strain for heterologous production. At least four kinds of transformation markers (*niaD*, *sC*, *adeA*, *argB*) are available in the strain NSAR1 [5], and the plasmids containing the biosynthetic genes with these markers are integrated into the genome as circular DNA. In most cases, however, it is difficult to regulate the genomic integration sites, and thus, in such transformants, the introduced genes are often not expressed, or the expression level is not uniformly regulated.

**Figure 6:**
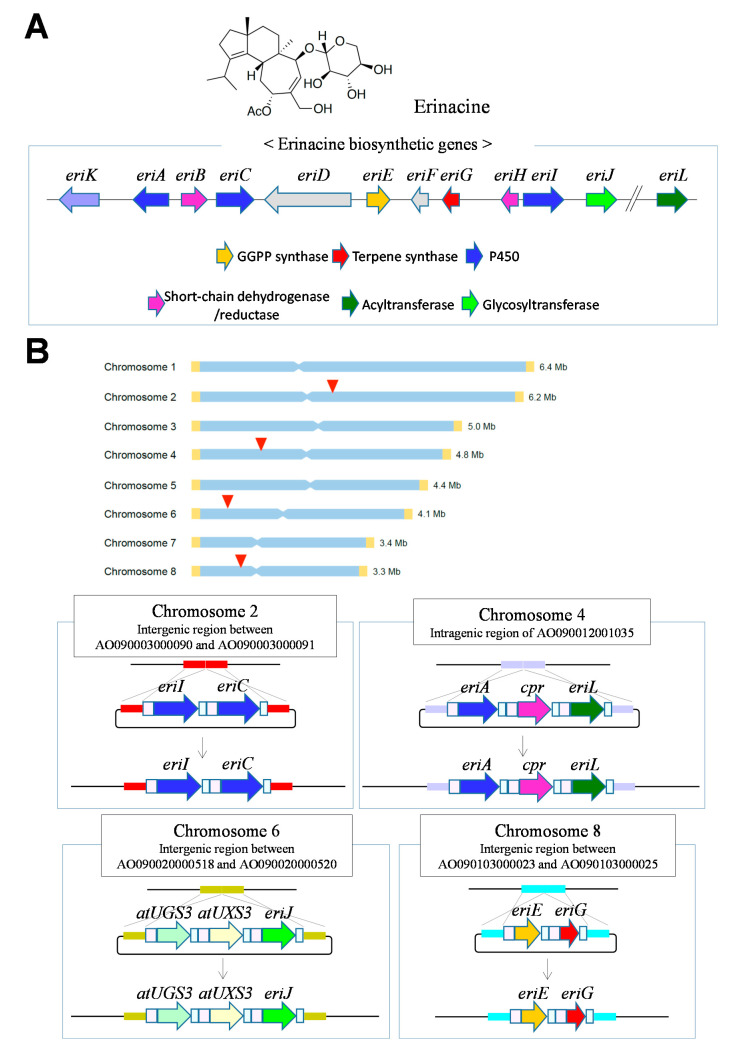
Heterogeneous production of natural product by *A. oryzae* using genome editing. (**A**) Erinacine and its biosynthetic genes in the basidiomycete *H. erinaceus*. (**B**) Targeted integration of 10 genes into four chromosomal sites (red arrowheads) for erinacine production by *A. oryzae*. Marker-free gene integration at each chromosomal site was due to highly efficient knock-in. *cpr*, a gene for cytochrome P450 reductase from *H. erinaceus* was used to improve the reaction efficiency of P450. *atUGS3* and *atUXS3* genes (cDNAs) derived from *Arabidopsis thaliana* were used for the supply of xylose.

Recently, biosynthetic genes have been efficiently introduced using the multiple-gene modification technology. Four genomic sites were identified where biosynthetic genes were integrated into high-producing strains obtained with a conventional plasmid [35,36]. Therefore, by targeting these genomic sites for knock-in by genome editing, biosynthetic genes can be introduced with high efficiency, and thus, gene expression is reliably obtained. Since the transformation marker is not integrated at the knock-in site, the genome editing plasmid can be used repeatedly, and, for example, the diterpen erinacine from the basidiomycete *Hericium erinaceus* was produced by integrating 10 biosynthetic genes into four genomic sites (Figure 6A,B) [36]. Under high homologous recombination efficiency due to the deficiency of LigD involved in NHEJ, a simultaneous knock-in into two genomic sites resulted in a highly-targeted integration with an efficiency of 66–100%.

Although the use of the conventional quadruple auxotrophic strain NSAR1 limits the number of genetic modification times, efficient multiple-gene modification technology by genome editing has the potential to infinitely increase the production level of natural products by metabolic engineering. As an example of endogenous metabolic modification in *A. oryzae*, the CRISPR/Cas9 system was used to delete *Aogld3* encoding a putative glycerol dehydrogenase, and production of the secondary metabolite kojic acid was decreased by affecting the expression of the genes *kojA* and *kojR* involved in kojic acid biosynthesis [37].

The CRISPR/Cas9 system was also used in heterologous protein production: deletion of the *Aooch1* gene involved in the generation of high-mannose *N*-glycan, and the *N*-glycan structure was expectedly altered in a human antibody produced by *A. oryzae* [38]. Further gene modification to express multiple heterologous glycotransferases using genome editing will allow the modulation of antibody activity by mimicking the mammalian *N*-glycan structure, strengthening the potential of *A. oryzae* as a platform of heterologous protein production.

## 6. Conclusions

Recent utilization of genome editing technologies, especially by the CRISPR/Cas9 system, has allowed highly efficient multiple-gene modifications in *A. oryzae*. It should be noted that there is no requirement to obtain an auxotrophic strain for transformation or to delete factors involved in NHEJ even in *A. oryzae* industrial strains, which had been quite difficult to genetically modify in the past.

In recent years, genome sequence information has become easily available due to next-generation sequencing, and it has become possible to link the characteristics and gene functions of each *A. oryzae* strain. Genome editing technologies can be used to accumulate new knowledge on the genetic characteristics of a wide variety of *A. oryzae* industrial strains. Even if the same gene is deleted, different phenotypes appear depending on the strains [39]. In heterokaryon incompatibility, any fused cells cannot exist depending on the combination of *A. oryzae* strains [40]. These aspects indicate the diversity of genetic characteristics in *A. oryzae* industrial strains. It is expected that genome editing technologies along with comparative genomics will facilitate gene function analysis for investigating individual industrial strains that have been specialized for brewing purposes, leading to functional development according to genetic characteristics. Indeed, the government of Japan has established guidelines for foods manufactured using genome-edited microorganisms by mandating a report that there must not be remaining foreign genes or their fragments in genomes [41]. Such foods do not require any safety reviews because the genome-edited microorganisms are developed to be indistinguishable from the naturally occurring mutant.

Additionally, an efficient multiple-gene modification technique using genome editing is effective for heterologous production of natural products as well as proteins. Genome editing is considered to be effective in the large-scale modification of metabolic pathways, which include a large number of enzyme genes and have robust properties that are compensated by other metabolic pathways. These technological innovations will accelerate synthetic biology, allowing programmable and tunable genome-scale engineering not yet seen in the breeding history of *A. oryzae*. Notably, genome editing technologies have dramatically improved gene modification efficiency in industrial strains of *A. oryzae*, paving the way for functional development in brewing and heterologous production.

## Data Availability

Not applicable.
